# Molecular epidemiology of HIV-1 subtype A in former Soviet Union countries

**DOI:** 10.1371/journal.pone.0191891

**Published:** 2018-02-01

**Authors:** Lazzat Aibekova, Brian Foley, Gonzalo Hortelano, Muhammad Raees, Sabit Abdraimov, Rakhmanbek Toichuev, Syed Ali

**Affiliations:** 1 Department of Biological Sciences, School of Science and Technology, Nazarbayev University, Astana, Kazakhstan; 2 Theoretical Biology and Biophysics Group, Los Alamos National Laboratory, Los Alamos, NM, United States of America; 3 Division of Pediatric Cardiac Surgery, Monroe Carell Jr. Children's Hospital, Vanderbilt University Medical Center, Nashville, Tennessee, United States of America; 4 Center for Disease Control and Prevention of AIDS, Astana, Kazakhstan; 5 National Academy of Sciences of the Kyrgyz Republic, Osh, Kyrgyzstan; 6 Department of Biomedical Sciences, Nazarbayev School of Medicine, Nazarbayev University, Astana, Kazakhstan; Public Health Agency of Canada, CANADA

## Abstract

While in other parts of the world it is on decline, incidence of HIV infection continues to rise in the former Soviet Union (FSU) countries. The present study was conducted to investigate the patterns and modes of HIV transmission in FSU countries. We performed phylogenetic analysis of publicly available 2705 HIV-1 subtype A *pol* sequences from thirteen FSU countries: Armenia, Azerbaijan, Belarus, Estonia, Georgia, Kazakhstan, Kyrgyzstan, Latvia, Lithuania, Moldova, Russia, Ukraine and Uzbekistan. Our analysis showed that the clusters from FSU countries were intermixed, indicating a possible role of transmigration in HIV transmission. Injection drug use was found to be the most frequent mode of transmission, while the clusters from PWID and heterosexual transmission were intermixed, indicating bridging of HIV infection across populations. To control the expanding HIV epidemic in this region, harm reduction strategies should be focused on three modes of transmission, namely, cross-border migration, injection drug use and heterosexual.

## Introduction

Regarding prevention and control of HIV infection, the global situation has been encouraging over the past few years. Recently, the rate of new HIV infections has slowed down, with an estimated annual number of new infections among adults remaining nearly static at about 1.9 million in 2015 [1]. During the same period, however, the annual estimate of new HIV infections in Eastern Europe and Central Asia increased by an alarming 57% [1]. In the Union of Soviet Socialist Republics (USSR), the first case of HIV infection was recorded in the 1980s [2]. Following that, further transmission of HIV-1 was recorded after the collapse of the USSR in 1991 [2, 3, 4]. During the years that followed, deterioration of socio-economic situation in the former Soviet Union (FSU) countries, coupled with visa-free cross-border movement, led to massive migrations within the Commonwealth of Independent States (CIS) countries [5, 6]. This cross-border migration facilitated, among other things, transmission of infectious diseases within the FSU countries. With large-scale production of opiates in the neighboring countries, and the southern Caucasus being a transit point for the trafficking of drugs between Europe and Asia, a remarkable rise in injection drug use was observed in the FSU countries [7]. Not surprisingly, the early HIV epidemic in the region originated amongst, and was initially driven by, people who inject drugs (PWID) [6, 8], and then spread into heterosexual population, where now the prevalence has surpassed that in PWID in most countries (Table 1) [6, 9, 10, 11, 12]. In addition to PWID and heterosexual communities, migrant workers, that are known to be an important transmission vehicle for infections, such as tuberculosis [3] and viral hepatitis [13], may also have played a role in HIV transmission within FSU countries [5].

**Table 1:** Prevalence of HIV-1 among key populations in 13 FSU countries.

Subtype	02_AG	03_AB	A	B	Others	Total
**Armenia**	3	1	235	9	2	250
**Azerbaijan**	-	1	37	3	-	41
**Belarus**	1	38	284	13	-	378
**Estonia**	10	5	80	54	1388	1538
**Georgia**	-	-	30	15	3	48
**Kazakhstan**	159	5	357	5	3	527
**Kyrgyzstan**	158	4	101	1	1	265
**Latvia**	4	-	623	163	7	797
**Lithuania**	1	93	80	59	15	248
**Moldova**	-	-	12	-	-	12
**Russia**	126	201	3493	364	745	4930
**Ukraine**	1	5	315	79	17	417
**Uzbekistan**	14	4	152	6	8	184

From the FSU countries, several studies have been reported the incidence and prevalence of HIV, and viral sequences have been sporadically deposited into global databases. However, a comprehensive study that takes into account the state of the epidemic and its patterns and modes of transmission within this region is still lacking. In the current study, using publicly available HIV sequences deposited during the years 1986–2015, we have analyzed the distribution of HIV-1 subtype A, the most prevalent subtype in the region, in thirteen FSU countries, namely, Armenia, Azerbaijan, Belarus, Estonia, Georgia, Kazakhstan, Kyrgyzstan, Latvia, Lithuania, Moldova, Russia, Ukraine and Uzbekistan.

## Methodology

To examine HIV transmission networks in FSU countries, 2705 publicly available HIV-1 subtype A pol sequences were downloaded from the Los Alamos HIV Sequence Database (data available as of April 3, 2016) for thirteen countries: Armenia (2009), Azerbaijan (2001–2002), Belarus (1997–2014), Estonia (2001–2010), Georgia (1998–2003), Kazakhstan (1997–2013), Kyrgyzstan (2002–2010), Latvia (1998–2008), Lithuania (1997–2007), Moldova (1997), Russia (1986–2015), Ukraine (1996–2012), and Uzbekistan (1999–2002). These sequences were aligned, edited, and used for the construction of phylogenetic tree as described elsewhere [14]http://journals.plos.org/plosone/article?id=10.1371/journal.pone.0093415. Briefly, the sequences were aligned and edited using the software *MEGA6*.*0* (www.megasoftware.net). The tree was drawn to scale, with branch lengths measured in the number of substitutions per site. Recombinant and duplicate sequences were identified using, respectively, *Recombinant HIV-1 Drawing Tool v2*.*1*.*0* and *ElimDupes* (www.hiv.lanl.gov), and were removed. Long branches on the tree were re-confirmed for their genotype and those found miscatalogued were eliminated from our study. After alignment and trimming, a 496-nucleotide long stretch of HIV *pol* sequence corresponding to HBX2 nucleotide 2748–3244 (GeneBank accession number K03455) was used for this analysis. To root the phylogenetic trees, 34 non-subtype A, Group M, reference sequences were used from Los Alamos HIV Sequence Database (www.hiv.lanl.gov). To construct the tree with risk group information, risk group data from the HIV Los Alamos website was linked to each sequence. The risk groups were then color-coded using either Rainbow tree (www.hiv.lanl.gov) or Figtree v1.4.2 (tree.bio.ed.ac.uk). To analyze transmission, relationship of branches was analyzed within clusters that were picked based on predominance of sequences from certain countries or high-risk groups.

Minimal data set for this study are available at: https://treebase.org/treebase-web/urlAPI.html

## Results

### Distribution of subtypes

We analyzed 2705 HIV subtype A *pol* sequences from thirteen FSU countries. The sequences were downloaded from HIV Los Alamos Database (www.hiv.lanl.gov) and from National Center for Biotechnology (NCBI). Phylogenetic analysis of these sequences showed that HIV-1 subtype A sequences were most predominant from all FSU countries in our study except for Kyrgyzstan and Estonia, where the most represented sequences were of CRF02_AG and subtype 06_cpx, respectively (Table 2). A considerable number of CRF02_AG sequences were also deposited from Kazakhstan. At low frequency, CRF03_AB and subtype B were also found in the FSU countries. Among other subtypes, circulating recombinant forms, and unique recombinant forms represented at low frequencies were CRF01/G, CRF01/AE, CRF02/A, CRF03/A, CRF06/A1, CRF06_cpx, CRF07/BC, CRFA1/B, and subtypes C, D, and G (Table 2).

**Table 2:** Distribution of HIV-1 subtypes in the HIV databases for thirteen FSU countries (www.hiv.lanl.gov).

	PLWH	HS (%)	PWID (%)	MSM (%)	MTC (%)	BT (%)	SW (%)	Pris (%)	Mig (%)
**Armenia**	3600	65	26	2.6	1.7	0.2	-	-	57
**Azerbaijan**	11000	31.9	56.9	1.2	1.5	0.02	0.7	5.8	38.4**
**Belarus**	35000	61.4	34.4	2.4	2.4	-	6.8	-	-
**Estonia**	9623	30	20	7	-	-	-	-	-
**Georgia**	9600	49	35	13	0.04	0.04	1.3	0.35	44.2***
**Kazakhstan**	23000	58	8.2	3.2	3.1	-	1.3	3.9	-
**Kyrgyzstan**	8100	62.3	12.4	6.3	0.04	-	2.2	7.6	-
**Latvia**	6800	38	21.3	8.07	1.15	-	-	-	-
**Lithuania**	2378	19.2	63.5	7.1	0.2	1.8	3.4	-	-
**Moldova**	18000	85.4	6.7	2.1	0.7	-	-	1.9	1.4****
**Russia**	742631	10.8	45.2	8	-	-	3.9	6.5	-
**Ukraine**	220000	69.2	24.3	22	3.8	-	-	-	-
**Uzbekistan**	33000	64.7	7.3	3.3	0.6	-	2.1	-	0.01

All data are cited from [16], except

*[24, 25]

**[26]

***[11], and

****[27] [3] [3].

Abbreviations: PLWH = People living with HIV, HS = Heterosexual pratices, PWID = People who inject drugs, MSM = men who have sex with men, MTC = Mother-to-child transmisison, BT = Blood transfusion, SW = Sex worker, Pris = prisoners, Mig = migrants

### Phylogenetic analysis

**Fig 1:**
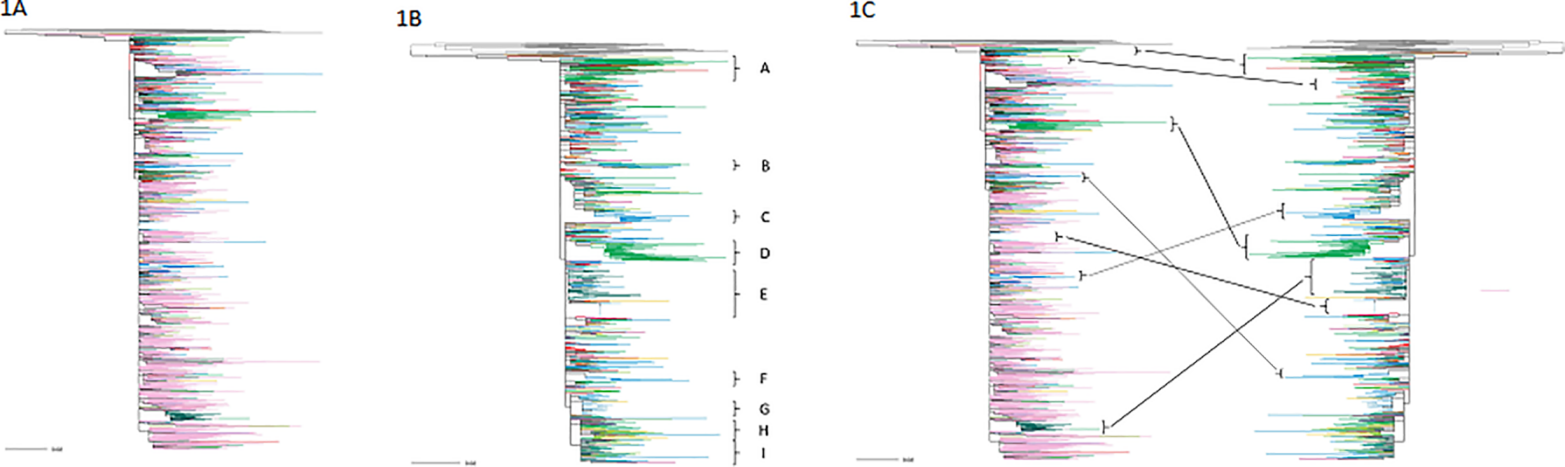
Phylogenetic networks of HIV subtype A in FSU countries. Analysis was performed by Maximum Likelihood (ML) method. A stretch of HIV *pol* sequence corresponding to HBX2 nucleotide 2748–3244 was used for this analysis, representing sequences from Armenia, Azerbaijan, Belarus, Estonia, Georgia, Kazakhstan, Kyrgyzstan, Latvia, Lithuania, Moldova, Russia, Ukraine and Uzbekistan, with (A) or without (B) Russia, represented by, respectively, yellow green, orange, green, deep pink, medium purple, blue, yellow, teal, hot pink, brown, light pink, red, and sky blue colored branches. The branches coded grey represent 34 outgroup non-subtype A reference sequences that were used to root the tree. **C)** Relationship between the clusters in trees A and B is indicated. Bootstrapped versions of trees A and B are provided as supplemental files labelled as, respectively, S1A and S1B Fig.

Since it was the most represented subtype from FSU countries, we focused our analyses on HIV subtype A. A phylogenetic tree was constructed to analyze the relationship between subtype A *pol* sequences in thirteen FSU countries: Armenia, Azerbaijan, Belarus, Estonia, Georgia, Kazakhstan, Kyrgyzstan, Latvia, Lithuania, Moldova, Russia, Ukraine and Uzbekistan, from the years 2009, 2001–2002, 1997–2014, 2001–2010, 1998–2003, 1997–2013, 2002–2010, 1998–2008, 1997–2007, 1997, 1986–2015, 1996–2012 and 1999–2002, respectively. 34 reference sequences of subtypes other than A were also retrieved from the Los Alamos Database and used as outliers to root the tree. The resulting tree clearly showed that the clusters from Baltic, Eastern European, Central Asian and Caucasus countries were inter-mixed with Russian strains, which indicated potentially overlapping transmission routes between Russia and other FSU countries (Fig 1A).

Since the number of sequences included from Russia was proportionally much higher, and because the sequence similarities overlapped among these countries, the phylogenetic clusters from other FSU countries became interspersed with Russian clusters. To get a clear idea of the transmission patterns within these countries, we constructed a second tree excluding all Russian sequences. This tree revealed a clearer picture of the phylogenetic relationship between the HIV sequences among the FSU countries (Fig 1B). A considerable intermixing of HIV-1 subtype A sequences from the 12 countries was observed. Interestingly, Ukraine sequence from the year 2001 formed ancestral node for the rest of the sequences (Fig 1B). Two monophyletic clusters were formed, each from Belarus (clusters A and D) and Kazakhstan (clusters B and C). The cluster from Kyrgyzstan (cluster H) showed presence of a few Armenia and Belarus sequences. Discrete clusters were also formed by sequences from Uzbekistan and Latvia. Uzbekistan sequences were observed intermixing with clusters F and G. The monophyletic cluster G observed at the bottom of the tree was mainly composed of the sequences deposited from Uzbekistan in 2002–2003. Kazakhstan sequences from 2002–2003, Kyrgyz sequences from 2009 and Latvia sequences from 2006 were also found in the Uzbekistan cluster G. Latvia sequences were found primarily in two distinct clusters (E and I), intermixed with sequences from Belarus and Caucasus countries (i.e., Armenia, Azerbaijan, and Georgia). This diverse representation of varied sequences within certain clusters possibly reflected migrant-associated HIV transmission within these countries. By comparing this tree with the one that included Russian sequences, it was possible to observe that the overall relationship among phylogenetic clusters from the six FSU countries remained consistent with or without the Russian sequences (Fig 1C).

### High risk group analysis

**Fig 2:**
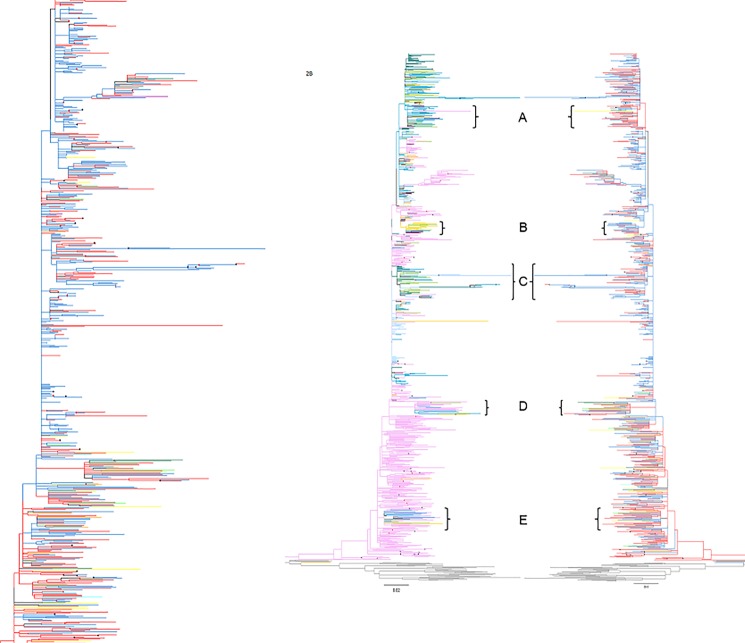
Analysis of HIV transmission among high risk groups in FSU countries. **A)** HIV-1 subtype A sequences from FSU countries were analyzed for high risk behavior. Only sequences with recorded high-risk labels were included in this analysis. Risk behavior information for each sequence was obtained from HIV Los Alamos Database. Red, orange, yellow, forest green, blue, turquoise, purple, brown, light pink, lime, and grey branches indicate, respectively, heterosexual, sexual transmission (unspecified type), MSM, mother-to-child, PWID, blood transfusion, homosexual, sex worker, bisexual, nosocomial, and reference sequences. **B)** To analyze risk behavior in the context of location, branches in the tree in 2A were re-colored based on country of origin (left), and the two trees were juxtaposed. Color key for the tree on the left is the same as that for Fig 1. Bootstrap values between 50–70 and ≤70 are shown, respectively, by red and black dots. For easy analysis, in the supplemental S2A Fig, clusters shown in Fig 2A have been magnified.

To explore the modes of HIV transmission among FSU countries, the tree in Fig 1A was further analyzed in the context of high-risk behavior. Fig 2A depicts a subtree that was constructed from the tree in Fig 1A with the branches colored according to the high-risk groups. To obtain a clear picture of transmission across high-risk groups, sequences for which risk group information was unavailable were discarded from this subtree. As a result, sequences from Lithuania and Estonia were excluded. This analysis revealed that sequences represented most from the FSU countries were heterosexual (HS) or PWID-associated, with the third most represented being homosexual (MSM) transmission (Fig 2A). An overall intermixing of sequences from PWID and hetero-/homo-sexual clusters was observed, possibly indicating bridging of HIV infection between the two groups. To analyze any possible association between countries and high-risk groups, the tree-branches in Fig 2A were re-colored based on county of origin, and the two versions of the tree were then juxtaposed (Fig 2B). In juxtaposed trees, cluster A with sequences mainly from Kazakhstan mixing with Armenia, showed predominance of HS transmission with some representation from PWID. Conversely, Cluster B, dominated by sequences from Kyrgyzstan, showed preponderance of PWID-associated sequences mixed with those from HS. Cluster C, with sequences from Kazakhstan and Armenia showed intermixing of PWID and HS-associated transmission. Both clusters D and E representing sequences mainly from Russia and Kazakhstan, interspersed with a few sequences from Kyrgyzstan, showed intermixing of sequences mainly from PWID and HS with a few that were MSM-associated.

## Discussion

In this study, using publicly available sequence data, we have analyzed the distribution of HIV-1 subtype A in thirteen FSU countries, namely, Armenia, Azerbaijan, Belarus, Estonia, Georgia, Kazakhstan, Kyrgyzstan, Latvia, Lithuania, Moldova, Russia, Ukraine and Uzbekistan. Focusing on HIV-1 subtype A, the most prevalent subtype in these countries, we performed phylogenetic analysis to determine the patterns of viral transmission in these countries. Our analysis revealed a considerable intermixing of HIV-1 subtype A sequences, possibly alluding to a high rate of transmigration-associated HIV transmission. Our analysis also indicated an association of HIV transmission with PWID and heterosexual populations.

Earliest reports of HIV transmission in FSU countries reflected repeated transmission of HIV-1 subtype A within the populations of these countries. For instance, at the beginning of the 21st century, the majority of people living with HIV in FSU were infected with an almost entirely homogeneous variant of HIV-1 subtype A, that later became known as A_FSU_ [2]. This was evidently a large-scale founder effect that, as a result of transmigration, led to the transmission of A_FSU_ in many FSU countries [2, 3, 4]. Additionally, CRF02_AG and Subtype B_FSU_ are reported to be the second most prevalent subtypes in former FSU countries [2], which was also the observation we made in our analysis. CRF02_AG was first reported in Uzbekistan (4.9%) in 1999–2000 [15]. The spread of CRF02_AG in FSU countries has been linked to the influx of the Central Asian labor forces and pathogenic fitness of the strain [2]. CRF02_AG is also thought to have been transmitted through transmigration most likely from Uzbekistan and Cameroon [6]. Subtype B, found to some extent in all FSU countries, is linked to labor migrants in Russia from Ukraine, and is thought to have been transmitted through unprotected sex among MSM, PWID and heterosexuals [2].

According to several reports, Ukraine is the place where HIV variant A_FSU_ originated and initially spread among PWIDs [12]. As can be seen from our analysis (Fig 1), HIV sequences from Ukraine did not only form ancestral node for, but also penetrated, the clusters from other FSU countries. Among the FSU countries, the burden of HIV epidemic is highest in Russia, with 742, 631 cases reported in 2015 [16]. There was a 13% increase in the incidence of new cases in 2014, compared to 2013 [16]. Owing to its relatively stronger economy, migration from other FSU countries to Russia is high, which might play a role in epidemiological bridging of HIV transmission between Russia and other FSU countries. In 2010, representation of migrant force in Russia was from Armenia (10.38%), and Azerbaijan (7.56%), Belarus (2.56%), Estonia (0.33%), Georgia (2.74%), Kazakhstan (14.54%), Kyrgyzstan (10.91%), Latvia (0.42%), Lithuania (0.22%), Ukraine (14.35%), and Uzbekistan (12.57%) [17]. Indeed, our phylogenetic analysis revealed a dense intermixing of HIV sequences between Russia and other FSU countries (Fig 1A and 1C)–a possible evidence of migrant-associated HIV transmission. Clusters from all FSU countries heavily intercalated with Russian sequences, indicating close sequence similarity among the HIV sequences from Russia and other FSU countries. This observation is in agreement with high rate of transmigration from other FSU countries to Russia, likely facilitating the bridging of HIV transmission networks in the region.

When phylogenetic analysis was performed without Russian sequences, a clearer picture of transmission networks was revealed with discrete clustering as well as intermixing of sequences observed between the FSU countries. A major high-risk group involved in HIV transmission among FSU countries is migrant workers (Table 1). Among the Commonwealth of Independent States, the share of migrant workers from the Central Asian countries amounts to 80–90% and from European part of CIS is over 50% [5], indicating high mobility of migrants within this region, that may possibly facilitate transmission of infectious diseases. Indeed, in our phylogenetic analysis, certain clusters of HIV sequences from FSU countries showed intermixing between countries that are known to involve in migrant exchange. For instance, monophyletic cluster G at the bottom of the tree almost entirely comprised subtype A sequences from Uzbekistan from 2002–2003, intermixing with a few Kazakhstan sequences from 2002–2003, Kyrgyz sequences from 2009 and Latvia sequences from 2006, which may indicate migrant-associated transmissions (Fig 1B). In the high-risk analysis, Kazakhstan sequences, mostly associated with PWID, HS, and MSM transmission were found intermixed with sequences from several FSU countries, including Armenia, Kyrgyzstan, and Russia. As Kazakhstan was one of the fastest economically growing country after the collapse of Soviet Union, in the period between 1997 and 2006, the traffic of migrants in Kazakhstan increased significantly, making Kazakhstan the country that received the ninth highest number of immigrants in the world [17]. This influx of migrants may have served as a route for HIV transmission in and out of Kazakhstan. Indeed, migrant movement between these countries has been reported [18,19], which may have led to cross-border HIV transmission. Another possibility is that bridging of infection might have occurred through Russia that served as a hub for migrant traffic across CIS. In fact, the main countries of destination for Kyrgyz and Armenia migrants are known to be Russia and Kazakhstan [19]. It is estimated that over 529 000 Kyrgyz labor migrants came to Russia in 2013 [19].

Drug trafficking routes that originate from opiate-producing central Asian countries, such as Afghanistan [20] crisscross in central Asia, with the “Northern route” of drug trafficking passing directly through the Central Asian region to Russia, the South Caucasus and then to Europe. Since the mid-1990s, there has been an important shift in drug trafficking volumes away from the Western towards the Northern route [21], with 20% of Afghan production now passing through Central Asia [22]. This is reflected in the cluster B (Fig 2B), where Kyrgyz sequences are found mixing with those from Georgia and Armenia; all three countries are located on this route. This increased drug trafficking may have played a major role in the early spread of HIV among PWID in FSU countries. However, according to recent reports, while in the past HIV epidemic in FSU countries was mostly concentrated among PWID, it is now bridging into heterosexual population (Table 1). In Kazakhstan, for the HIV infections reported in 2002, PWID-related transmissions accounted for more than 90% of the total [23]. Following the initial massive founder effect, HIV-1 subtype A is now reported to show gradual mutational drifts, possibly owing to the switching of the route of transmission from PWID to heterosexual population, and also due to the mobility of FSU citizens in to countries outside the Central Asian region [2]. Analysis of high risk behavior we performed in conjunction with phylogeny did, indeed, show that in the HIV sequences we studied the infection were mainly associated with PWID, while indications of bridging from this to the heterosexual, and to some extent to homosexual, population were also observed (Fig 2A & 2B).

## Conclusion

During that last decade, HIV cases have continued to rise in the FSU countries. The phylogenetic analysis we present here indicates that HIV transmission in these countries has occurred mainly through PWID communities and is now emerging among the hetero- and homo-sexual population. Additionally, our phylogenetic analysis shows an intermixing of phylogenetic clusters that are likely to have resulted from transmigration within FSU countries. It therefore follows that the high-risk groups that need to be the focus of harm reduction policies are PWID, heterosexual population and migrant labor. It should be pointed out that the observations presented here were limited by the HIV sequences available to us through public databases. We were thus able to analyze sequences that were deposited only up to 2015. To get a more realistic picture of the evolving HIV epidemic in FSU countries, prospective studies should be designed to analyze HIV sequences periodically from the high-risk groups in this region.

## Supporting information

S1 Fig **A and B.** Full-scale version of the trees in Fig 1 with accession numbers and bootstrap values indicated. Bootstrap values between 50–70 and ≤70 are shown, respectively, by red and black dots.
(TIF)

S2 Fig For easy reference and analysis clusters in Fig 2 are shown at high resolution and with accessions numbers included in the labels. In the full version of the tree (left), bootstrap values between 50–70 and ≤70 are shown, respectively, by red and black dots.
(TIF)
