# Supplementary figures and images for: Molecular epidemiology of HIV-1 subtype A in former Soviet Union countries
Source: PLoS One. 2018 Feb 1;13(2):e0191891. doi: 10.1371/journal.pone.0191891 (PMC5794106; doi:10.1371/journal.pone.0191891)

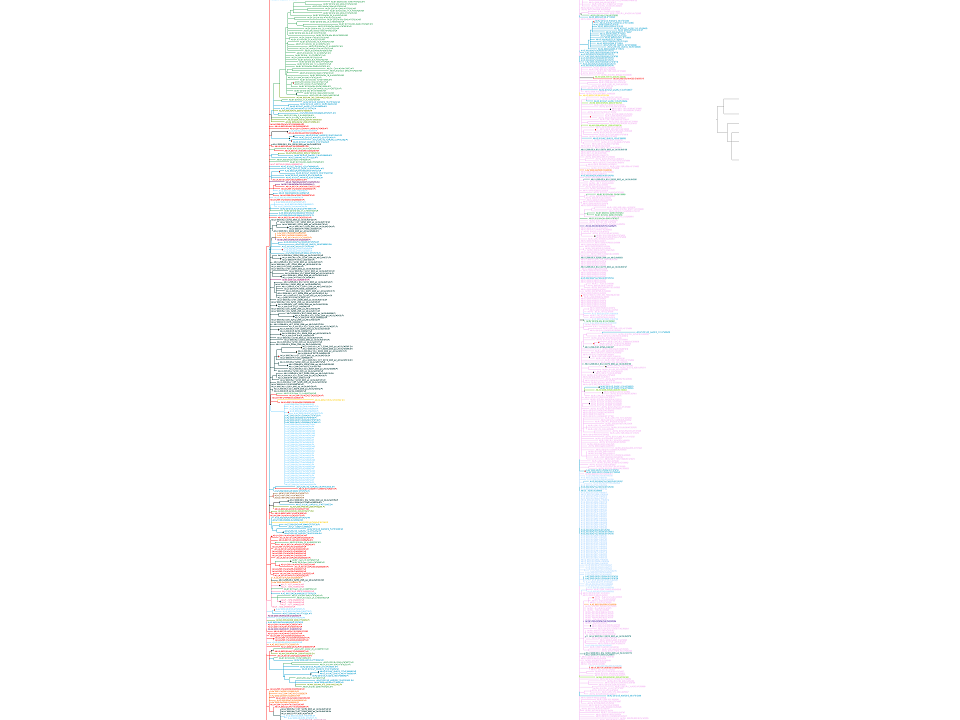

Supplement: S1 Fig — A and B. Full-scale version of the trees in Fig 1 with accession numbers and bootstrap values indicated. Bootstrap values between 50–70 and ≤70 are shown, respectively, by red and black dots. (TIF) [file pone.0191891.s001.tif]

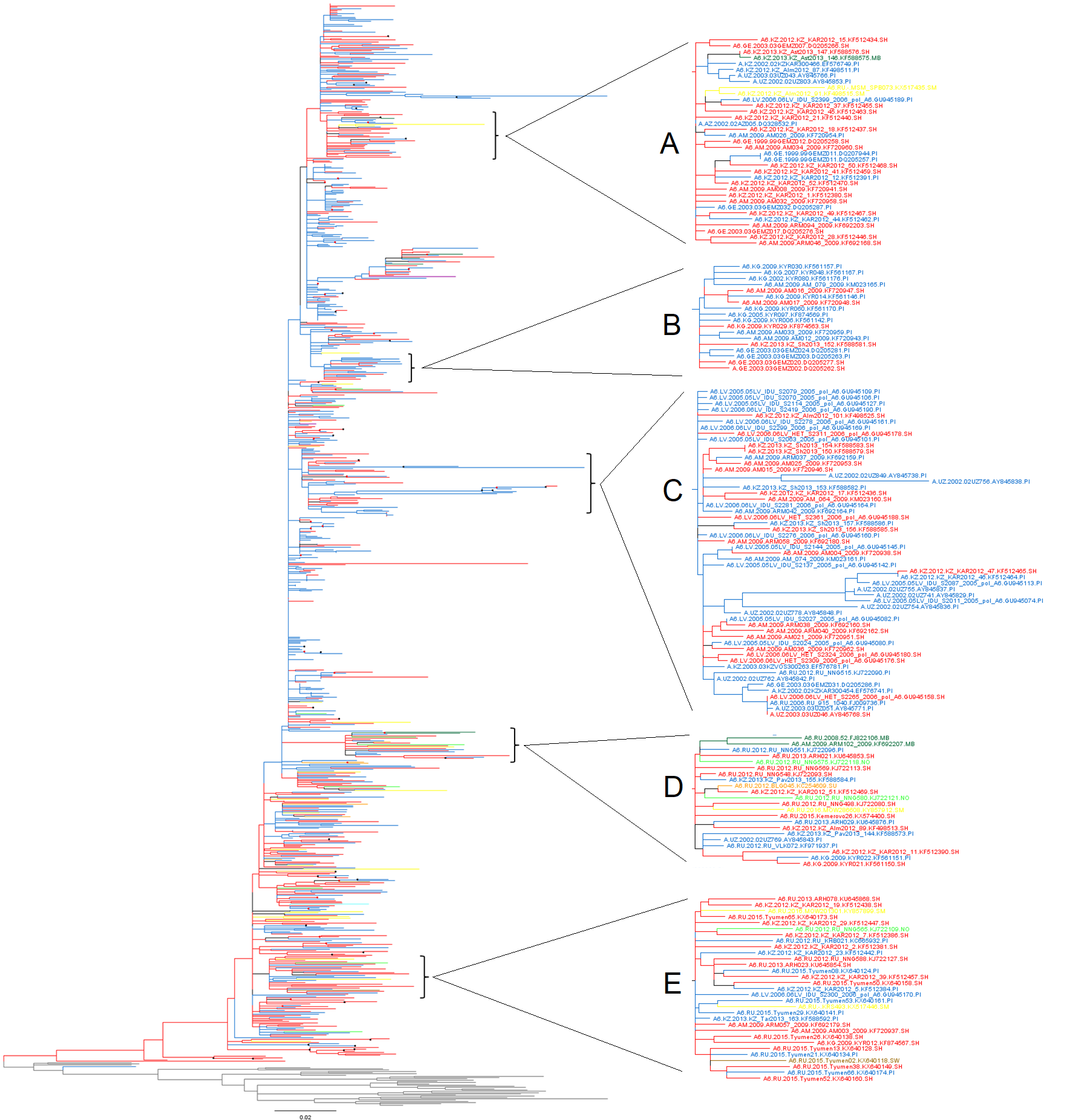

Supplement: S2 Fig — In the full version of the tree (left), bootstrap values between 50–70 and ≤70 are shown, respectively, by red and black dots. (TIF) [file pone.0191891.s002.tif]
